# Low-Density InGaAs/AlGaAs Quantum Dots in Droplet-Etched Nanoholes

**DOI:** 10.1021/acs.nanolett.5c04426

**Published:** 2026-01-15

**Authors:** Saimon F. Covre Da Silva, Ailton J. Garcia, Maximilian Aigner, Christian Weidinger, Tobias M. Krieger, Gabriel Undeutsch, Christoph Deneke, Ishrat Bashir, Santanu Manna, Melina Peter, Ievgen Brytavskyi, Johannes Aberl, Armando Rastelli

**Affiliations:** † Institute of Semiconductor and Solid State Physics, 27266 Johannes Kepler University Linz, Altenberger Straße 69, 4040 Linz, Austria; ‡ Instituto de Física Gleb Wataghin, 28132 Universidade Estadual de Campinas (UNICAMP), 13083-859 Campinas, Brazil; § Department of Electrical Engineering, 28817 Indian Institute of Technology Delhi, Hauz Khas, New Delhi, Delhi 110016, India

**Keywords:** molecular beam epitaxy, local droplet etching, semiconductor quantum dots, single photon sources

## Abstract

Over the past two decades, epitaxial semiconductor quantum dots (QDs) have demonstrated very promising properties as sources of single and entangled photons on-demand. Among different growth methods, droplet etching epitaxy has allowed the growth of almost strain-free QDs, with low and controllable surface densities, small excitonic fine structure splitting (FSS), and fast radiative decays. Here, we extend the technique to In­(Ga)As QDs in AlGaAs, thereby increasing the achievable emission wavelength range beyond that accessible to GaAs/AlGaAs QDs while preserving some of the key advantages of this growth method. We observe QD densities of ∼0.25 μm^–2^, FSS values as small as 3 μeV, and short radiative lifetimes of ∼300 ps, while extending the achievable emission wavelength to ∼900 nm at cryogenic temperatures. We envision these QDs to be particularly suitable for integrated quantum photonics applications.

## Graphical abstract

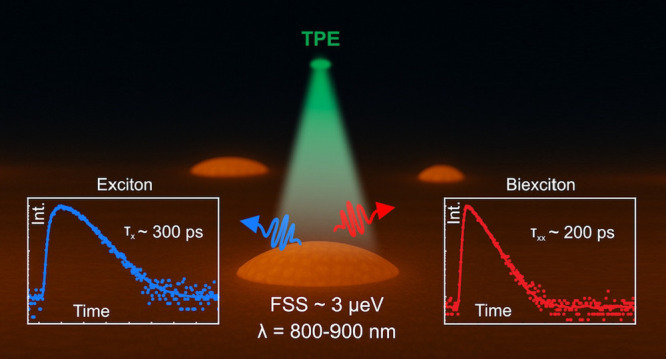

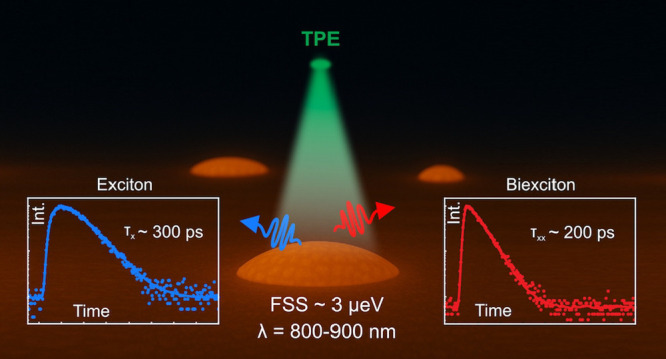

Quantum technologies, especially quantum communication^1–4^ and photonic quantum simulation,^5,6^ require advanced sources of quantum light. These include sources based on spontaneous parametric down-conversion (SPDC),^7^ trapped atoms,^8^ and semiconductor quantum dots (QDs).^9^ QDs are attractive due to their high quantum efficiency and brightness,^10^ as well as near-unity photon indistinguishability^11^ and entanglement fidelity.^12^ Offer an “on demand” operation with sub-Poissonian photon counting statistics,^13,14^ while also allowing to fine-tune the emission characteristics across a broad spectral range.^15^ More specifically, InGaAs QDs obtained by the Stranski-Krastanow (SK) growth mode of InAs on GaAs(001) have been used for many pioneering proof-of-principle experiments^16^ and are now commercially available.^17,18^ These developments have been enabled by the integration of QDs in photonic nanostructures and devices.^19^ One of the challenges with InGaAs SK QDs is the difficulty of growing them with a surface density low enough to optically address single QDs over a full GaAs wafer.^20–23^ In addition, inhomogeneous In alloying leads to large excitonic fine-structure splitting (FSS)^24^ and also a noisy nuclear spin environment,^25,26^ possibly limiting their performance as sources of polarization-entangled photon pairs.^27^ Furthermore, the typical excitonic radiative lifetime of 1 ns,^28^ as well as the presence of wetting-layer states,^29^ lead to a significant dephasing, limiting their utility in quantum applications.^29,30^ Although InGaAs QDs with short radiative lifetime (high oscillator strength) can be obtained by InGaAs deposition on GaAs^31^ or by InAs deposition at low growth rates^21^ followed by ex-situ rapid thermal processing,^32–34^ these approaches are either accompanied by difficulties in controlling the QD density or by an additional high-temperature processing step. In contrast, QDs grown by filling local droplet-etched (LDE) nanoholes with GaAs, pioneered by Heyn et al.^35^ and further simply referred to as GaAs QDs, have overcome some of these limitations. Allow for the growth of QDs with higher symmetry and low surface density, improved ensemble homogeneity, and higher oscillator strengths compared to SK QDs.^36^ These superior properties are reflected in a low surface density of approximately 0.2 μm^–2^, an average FSS of 3 μeV and below^36^ and a short radiative lifetime in the order of 200 to 250 ps,^37^ making these QDs a promising alternative to SK QDs. When embedded in diode structures, GaAs QDs routinely display emission line widths close to the transform limit.^38–40^ However, the longest wavelength achievable by GaAs QDs is inherently limited to the emission wavelength of free excitons in GaAs (815 nm at typical cryogenic temperatures of around 5 K). Longer wavelengths are desirable for QDs embedded in nanophotonic structures, where the impact of fabrication imperfections, as well as scattering and absorption losses in Al­(Ga)As scale with the wavelength.^41^ In addition, experimental setups and technologies designed for conventional InGaAs SK QDs would benefit from QDs emitting in similar wavelength ranges. Recently, the advantages of LDE for producing nanostructures with low surface density have been applied to various systems, such as AlGaSb/InGaSb^42,43^ and, in combination with SK growth, to obtain SK QDs with low density over full wafers^44,45^ also with emission in the telecom O-band.^46^ In this work, we take a different approach. Based on an established method for etching nanoholes in AlGaAs using molecular beam epitaxy (MBE), we deposit a layer of In_*x*_Ga_1–*x*_As with a nominal In content of *x* = 0.1–0.4 to fill the nanoholes. Surface characterization by atomic force microscopy (AFM) as well as photoluminescence (PL) imaging show a low QD density (∼0.25 μm^–2^; see the Supporting Information), suitable for single spectroscopy. Optical μ-PL measurements show very low FSS values (as small as 3 μeV), radiative lifetimes (around 300 ps), and emission line widths (13 μeV), that are comparable to the values reported for GaAs QDs in intrinsic material. Hence, at least for experiments and applications for which residual strain and alloy disorder are tolerable, such as those relying on using QDs as quantum light emitters, we can maintain the beneficial properties of GaAs QDs and extend the emission wavelength range from below the GaAs bandgap to values usually reachable by InGaAs QDs either treated with partial capping and annealing^47^ or by rapid thermal treatment.^32,48^

All studied samples were grown on Si-doped GaAs (001) substrates by using a solid-source molecular beam epitaxy system (MBE-Komponenten GmbH) equipped with an As-cracker source. Figure 1(a) shows a schematic illustration of the sample structure. Initially, a buffer layer is grown on top of the GaAs substrate at 590 °C, followed by a 200 nm thick Al_0.33_Ga_0.67_As layer that serves as the bottom barrier for the QDs. In the following step, we employed LDE to create ∼8 nm deep nanoholes with Al droplets. A more detailed description of the LDE process and GaAs QD fabrication can be found in ref^36^ and in the Supporting Information. The substrate is then cooled to 495 °C to limit In desorption^49^ and 1 nm of nominal In_*x*_Ga_1–*x*_As is deposited, followed by a 30 s annealing step, a 0.5 monolayers (ML) GaAs cap and another 200 nm layer of Al_0.33_Ga_0.67_As, serving as the top barrier. Subsequently, the etching and filling process is repeated on the surface for AFM measurements. Note that the surface QDs are optically inactive due to the presence of a large density of nonradiative centers. Figure 1(b) shows a PL image collected at 10 K of the sample with *x* = 0.3. Individual QDs are visible as well-separated bright spots. From these and similar images, we estimate that the QD density varies between approximately 0.2 and 0.3 μm^–2^ (see the Supporting Information). Similar densities, which are solely determined by the initial Al droplet etched nanoholes, are observed also in the other samples discussed in this work. Achieving this distribution is straightforward using the LDE method compared with the SK method. We note that fluctuations in the QD brightness could stem from differences in diffusion and capture of charge carriers, which are photogenerated in the AlGaAs barriers, as well as possible differences in quantum efficiency. Figure 1(c) shows a high-resolution 1 × 1 μm^2^ AFM scan of the sample with *x* = 0.4, clearly revealing a filled QD structure after 1 nm of InGaAs deposition. In the surrounding of the formed mound, the surface is characterized by 2D terraces typical of the growth of an InGaAs layer through MBE.^50^ The material diffuses across the surface and fills the nanoholes completely. The mound is elongated along the [1–10] direction, with an approximate length of 300 nm, a width of 120 nm and a height of 1.5 nm. The mound formation and evolution arise from the anisotropic diffusion of material on top of the AlGaAs surface,^22^ which has the tendency to fill the etched holes according to their morphology.^51^ Figure 1(d) represents a 20 × 5 μm^2^ AFM overview image of the same sample (with *x* = 0.4), showing no evidence of SK QD formation over a large area. The bright spots correspond to the mounds formed after nanohole filling, as shown in Figure 1(c). The absence of SK island formation in the current and related samples is attributed to the low amount of deposited InGaAs (1 nm), which is below the critical thickness (approximately 2 nm for In_0.4_Ga_0.6_As/Al_0.33_Ga_0.67_As)^52^ required for 3D island formation for the used In concentrations. The fact that nanoholes (bottom surface of the InGaAs QD) are produced in the same nominal way and that the top InGaAs surface is similar for all values of *x* means that the QDs studied here have nominally the same shape and size but different stoichiometry. This level of control is not available for SK QDs, where the shape, size, and compositions are tightly linked.^53^

**Figure 1:**
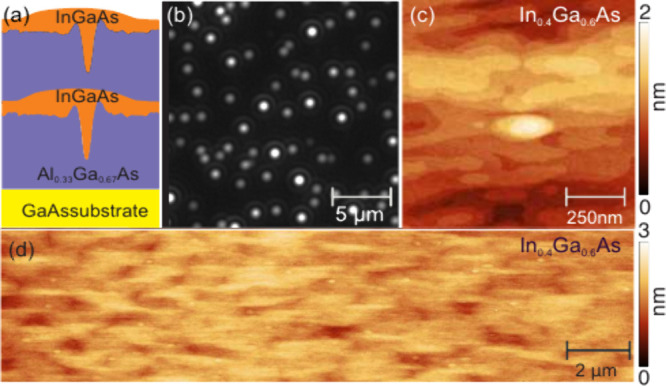
(a) Schematic illustration of the sample structure using AFM linescans (the vertical scale of the nanoholes and mounds is exaggerated). (b) Cryogenic PL microscopy image, obtained by illumination of the sample with *x* = 0.3 with incoherent blue light, showing the PL of single QDs with a density of ∼0.20(2) μm^–2^. (c) AFM image illustrating the morphology of a single AlGaAs nanohole filled by depositing 1 nm of In_0.4_Ga_0.6_As followed by 30 s annealing. (d) 20 × 5 μm^2^ overview AFM image of the same sample as in (c), showing no evidence of SK QD formation.

For the basic optical characterization of single QDs, a confocal μ-PL setup with a 50× microscope objective with 0.42 NA is used. Measurements are performed with samples at 10 K and with above-band gap excitation (533 nm diode laser). A separate confocal μ-PL setup with a 0.65 NA objective and a tunable, pulsed titanium-sapphire laser is employed for time-correlated single-photon counting measurements, coherence time measurements, and autocorrelation measurements. These results are presented in Figures 3–5.

Figure 2 shows the results of μ-PL measurements on randomly chosen single QDs. Compared to GaAs QDs, the emission wavelength of the neutral exciton in these QDs is red-shifted. This shift increases with the In content in the QD, allowing for emission wavelength control from 780 nm up to 900 nm by varying the nominal In concentration during growth.

**Figure 2:**
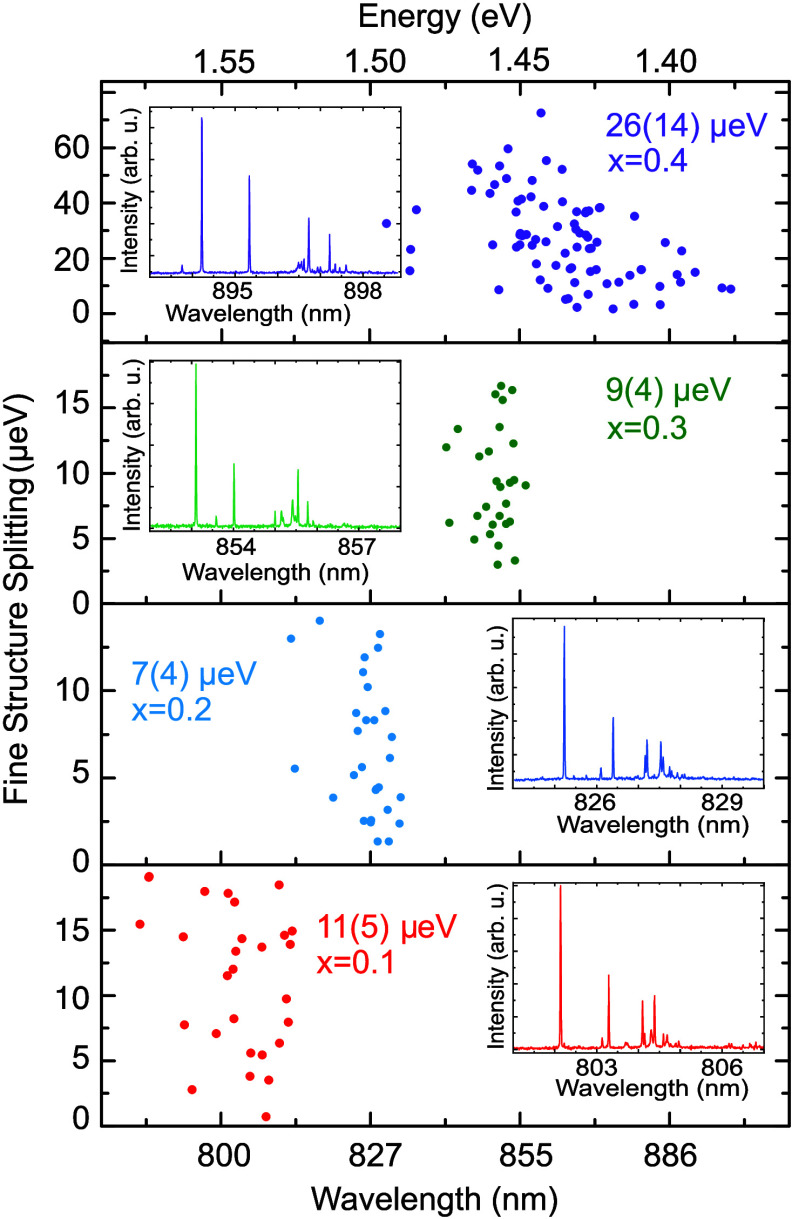
Scatter plots of excitonic FSS and emission wavelength of InGaAs QDs obtained by filling AlGaAs nanoholes by deposition of In_*x*_Ga_1–*x*_As with different values of *x*. From the bottom to the top, panels show results for *x* = 0.1, 0.2, 0.3, 0.4. The inset in each panel shows a representative spectrum of an isolated single QD with the respective nominal In concentration.

For each *x* value, a representative emission spectrum is shown in the respective inset. The spectra qualitatively resemble those of GaAs QDs^36^ with the dominant emission stemming from the neutral exciton (X) recombination and additional charged exciton lines at longer wavelengths.^54^ For the samples with *x* = 0.1, 0.2, and 0.3, 30 QDs were measured, showing a wavelength distribution with a standard deviation of less than 8 nm. On the sample with *x* = 0.4, a higher spread in wavelength distribution with a standard deviation of above 20 nm was observed, so 80 QDs were measured for better statistics. Most of the observed QDs have an X line width below the setup resolution limit of about 0.02 nm.

To gather information on the alloy composition of the obtained QDs, we estimated the ground-state optical transitions of InGaAs-filled nanoholes by approximating these QDs as 8 nm thick In_*x*_Ga_1–*x*_As quantum wells (QWs) with Al_0.33_Ga_0.67_As barriers and by calculating the confined electronic energy levels within the envelope function approximation using single band and eight-band k·p calculations. The calculated transition energies were found to be significantly red-shifted in comparison to the experimentally measured values, suggesting that the actual In within the QDs is lower than the nominal values set in the growth recipe. As an example, for the sample with a nominal In fraction *x* = 0.4, we experimentally find an emission wavelength near 900 nm, which corresponds to a QW with an *x* of only 0.15. Although a direct experimental proof is not yet available, we qualitatively attribute this discrepancy to In surface segregation leading to a lower than nominal incorporation of In into the nanoholes and a net loss of In during the temperature ramp step preceding the growth of the AlGaAs layer on top of the InGaAs layer. Further details on the model and growth are provided in the Supporting Information.

Next, the excitonic FSS is assessed via polarization-resolved measurements. The distribution of FSS has average values of 11(5) μeV for *x* = 0.1, 7(4) μeV for *x* = 0.2, 9(4) μeV for *x* = 0.3, and 26(14) μeV for *x* = 0.4. We note here that within the sample with *x* = 0.4 we also find QDs with considerably smaller FSS of less than 3 μeV. In general, this sample exhibits a broader distribution in both wavelength and FSS compared to the other samples. Since we expect the shape of the QDs to be dictated by the nanohole shape, which is nominally the same for all values of *x*, we ascribe the broadening to compositional and strain fluctuations associated with the presence of In. By tuning the growth parameters during InGaAs deposition and subsequent annealing and capping, we expect that some improvement is possible. Although the spread in emission wavelength of InGaAs/AlGaAs QDs is larger than in GaAs QDs, the ordering of excitonic complexes is comparable, which is usually not the case in SK QDs.^55^

To gain further insight into the optical properties of our InGaAs/AlGaAs QDs, we investigated the sample with *x* = 0.4 using different excitation schemes. Pulsed two-photon resonant excitation (TPE) enables coherent population of the biexciton state (|XX⟩) followed by the emission of XX and X photons via a radiative cascade.^56^ To maximize the emission intensity by limiting blinking and charge fluctuations, we use an additional continuous wave diode laser with above energy emitting at 532 nm. The inset in Figure 3(a) displays a spectrum showing XX and X emission lines, as well as partially filtered laser light in between. In Figure 3(b), the power dependence of the intensity of XX and X lines is shown, exhibiting well-defined Rabi oscillations and confirming the coherent control of the |XX⟩ state. Figure 3(a) shows the results of the time-correlated single-photon counting measurements. Lifetimes τ_XX,TPE_ = 197(11) ps and τ_X,TPE_ = 318(23) ps are obtained by performing a single and double-exponential fit of the data, respectively, convoluted with the instrumental response function. The values are approximately three times shorter than those measured in SK-grown InGaAs QDs^28^ emitting in the same wavelength range are comparable to SK-QDs treated with rapid thermal annealing^57^ and can be further reduced by embedding the QDs in optical microcavities, such as defects in photonic crystals,^58^ micropillars,^11^ or circular Bragg resonators.^12,59,60^

**Figure 3:**
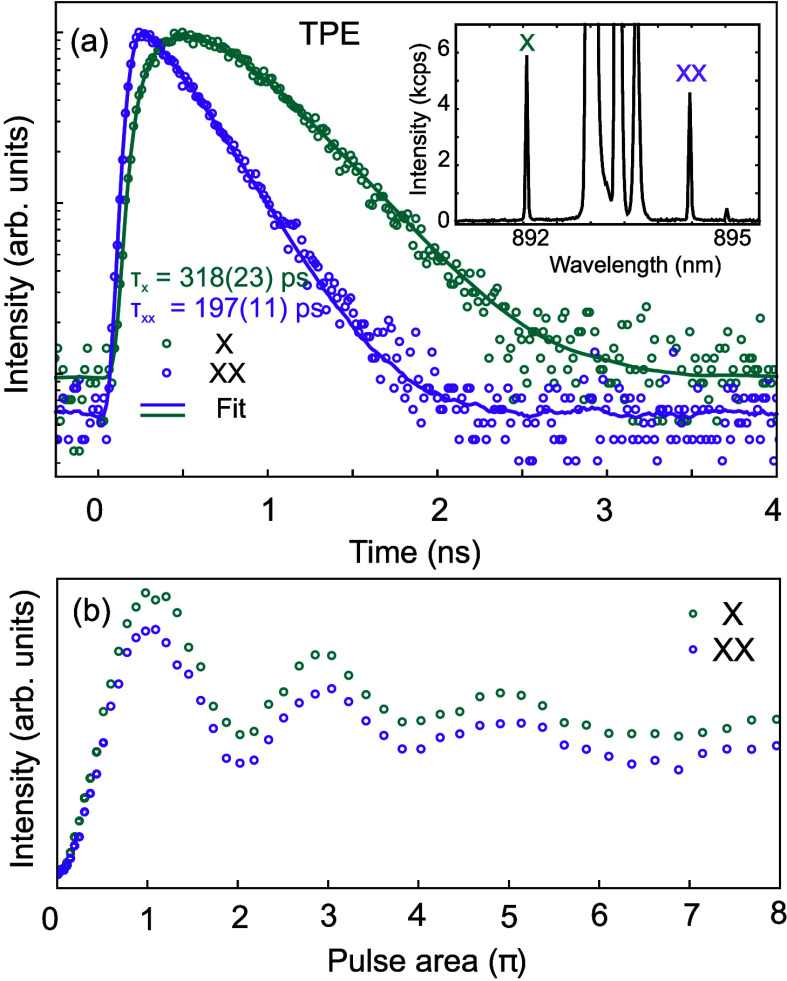
(a) Time-correlated-single-photon-counting measurement of the decay of |X⟩ (green) and |XX⟩ (purple) confined in a QD in the sample with *x* = 0.4 after coherent TPE through a laser with ≈ 893 nm wavelength, < 10 ps pulse duration, and 80 MHz repetition rate. The inset shows the corresponding PL spectrum (residual laser light is seen in between the X and XX lines). (b) Excitation-power-dependent behavior of peak intensity versus the excitation pulse area for X (green) and XX (purple) emission for the same QD, showing clear Rabi oscillation.

To provide insights into carrier capture and relaxation times via phonon-mediated processes, we extend the time-correlated single-photon counting of X photons to incoherent excitation techniques. In fact, it is known that GaAs QDs obtained by the LDE method are characterized by slow interlevel relaxation, which often masks the true radiative decay when excitation is performed using laser energies above-bandgap or resonant to QD excited states.^37^ Furthermore, incoherent excitation is easier to implement than resonance fluorescence, because of straightforward spectral laser filtering. To illustrate the QD level structure, for optimizing excitation schemes, a spectrum under high-power above-bandgap excitation of a representative QD with *x* = 0.4 is shown in Figure 4(a). From the spectral positions of the different emission bands, an energy separation of approximately 26 meV (16 nm) between the s-shell and p-shell can be extracted. This value is approximately twice what is observed in common GaAs QDs with emission wavelength around 780 nm^37^. Since the AlGaAs nanoholes are fabricated following the same procedure and the amount of material used for nanohole filling is also similar, we expect the sizes and shapes of the QDs studied here to be similar to those of GaAs QDs. We thus attribute the difference in shell spacing to the presence of In, which not only decreases the average energy bandgap of the QD material but also increases the conduction-band offset and decreases the carrier effective masses, thus increasing the particle confinement energies.

**Figure 4:**
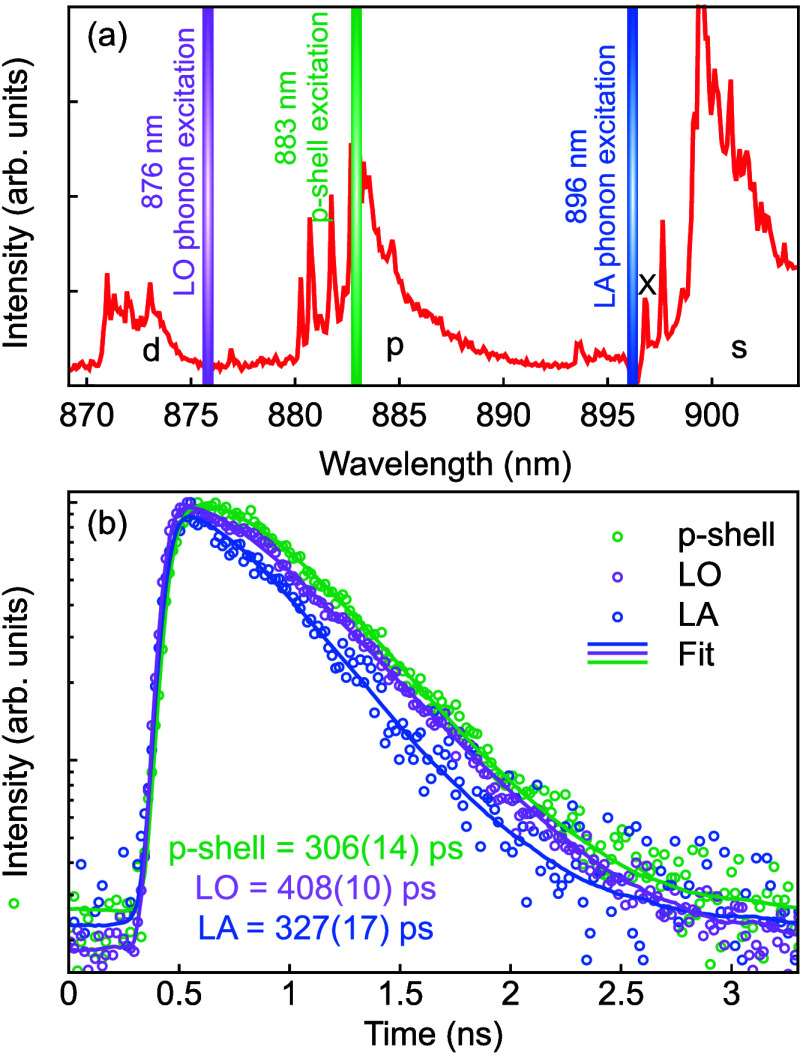
(a) PL spectrum of a QD in the sample with *x* = 0.4 under high-power above-bandgap excitation, showing the position of the s-, p-, and d-shell. (b) Time-correlated single-photon counting conducted under various excitation schemes (LA-phonon, p-shell and LO-phonon). The measurements were performed on X photons (897 nm).

Commonly used incoherent excitation techniques are longitudinal acoustic phonon-assisted (LA), p-shell excitation, and longitudinal optical phonon-assisted (LO) excitation. We employ these by tuning a pulsed laser to the respective wavelengths shown in Figure 4(a). For each excitation wavelength, we recorded time traces, shown in Figure 4(b), which are fitted to extract the total lifetime of the excited state. For LA excitation, we expect the phonon interaction to be fast compared to the lifetime of the exciton. Data are therefore well fitted through a single exponential fit, resulting in τ_*X*,*LA*_ = 327(17) ps. For p-shell excitation, carriers first need to relax to the s-shell before X emission can occur. In general, this relaxation time can not be neglected and is taken into account by performing a double exponential fit. We extract a relaxation time into the s-shell of 255(18) ps and an exciton lifetime of τ_*X*,*p*_ = 306(14) ps. Ideally, LO excitation also leads to a single exponential decay of the X photon counts. From the fit, we extract a lifetime of τ_*X*,*LO*_ = 408(10) ps, which is, however, larger than the values obtained by the other excitation techniques. We assume that additional states are excited by the laser, which lead to a mixture of different decay paths. The delayed decay we observe compared to the data obtained under LA-assisted excitation is consistent with this interpretation. A comparison to GaAs QDs indicates that the transition from p- to s-shell is significantly faster for the InGaAs QDs studied here.^37,61^ We ascribe this observation to the dependence of the relaxation time on the s-p energy separation.^62,63^

To further assess the optical quality of our QDs, we determine the time-averaged coherence time of X photons emitted by a single QD in the sample with *x* = 0.4 under above-bandgap excitation using a 532 nm diode laser. To this end, the interference visibility was measured in a Michelson interferometer for different relative time delays among the two interferometer arms. The results, shown in Figure 5(a), are fitted with the Fourier transform of a Lorentz profile. We extract a line width of Δ*E*_L_ = 13.0(4) μeV (coherence time *t*_*L*_ = 101(3) ps), which, compared to the Fourier limit (Δ*E*_F_ = 2.2(1) μeV) is broadened by a factor of 5.9(5). This value is comparable to that typically observed in state-of-the-art GaAs QDs and is likely dominated by charge noise, which can be mitigated by embedding the QDs in a p-i-n diode structure,^38^ as well as zero-phonon line broadening due to non ideal sample cooling.

**Figure 5:**
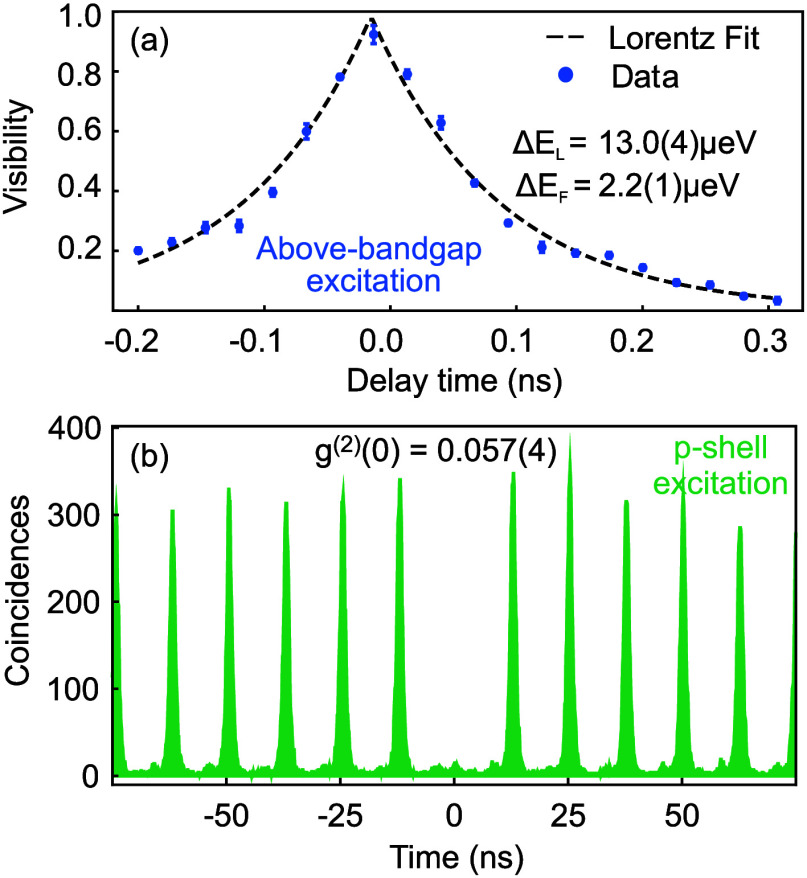
(a) Interference fringe contrast of neutral exciton photons from a QD in the sample with *x* = 0.4 as a function of time delay between the two branches of a Michelson interferometer under above-bandgap excitation at 10 K. The Fourier limit Δ*E*_*F*_ was estimated from the lifetime obtained through p-shell excitation. (b) Autocorrelation histogram recorded using the X emission of another QD in the same sample under p-shell excitation at 10 K.

Eventually, the single photon emission characteristic was assessed by using a Hanbury-Brown-Twiss (HBT) setup with two Excelitas single photon counting modules (SPCM-AQRH12). The resulting histogram, collected under p-shell excitation, is shown in Figure 5(b). Clear antibunching can be observed, and a *g* ^(2)^(0) value of 0.057(4) is obtained. We attribute this finite value to the background of the laser used for charge stabilization, as well as laser reflections on optical elements, that are also visible as mounds between main correlation peaks.

In conclusion, we have shown that it is possible to obtain high-quality InGaAs QDs in LDE-etched AlGaAs nanoholes and adjust their emission wavelength by controlling the nominal In concentration while retaining some of the favorable properties of established GaAs/AlGaAs QDs. These InGaAs QDs exhibit the same low surface density as GaAs QDs, making them ideal for devices based on single QDs. In particular, we expect the longer emission wavelength compared to GaAs QDs to be advantageous for integrated quantum photonics as longer wavelengths are associated with lower optical losses and relaxed fabrication tolerances. Additionally, it may become possible to interface these QDs with Cesium atom-based quantum memories not only via the *D*_1_ transitions at 895 nm, but also using the *D*_2_ lines at 852 nm, that are usually inaccessible to conventional InGaAs/GaAs SK QDs. The QDs presented here also feature a small excitonic FSS, which is useful for the generation of polarization-entangled photon pairs. Although the FSS presented in this work, especially for the sample with *x* = 0.4, is higher relative to recent works using GaAs QDs,^36^ further optimization of the growth process is still possible. The observed excitonic radiative decay times of ∼ 300 ps are close to those observed for GaAs QDs and faster compared to SK grown QDs emitting in the same wavelength range. By making use of the compatibility of the presented In­(Ga)As QDs in droplet-etched nanoholes with photonic structures, we envision that in the weak coupling regime, their radiative lifetimes could be further reduced to values around 10 ps via an already demonstrated Purcell enhancement of 25.^60^ Such ultrashort lifetimes would represent an important milestone toward next-generation quantum light sources that are robust against dephasing effects and can be driven at rates well above 10 GHz. In addition, assuming negligible contribution of nonradiative decay processes, the large estimated value of the oscillator strength of ∼35,^64^ makes our QDs attractive for achieving the strong coupling regime in microcavities.^31,65–67^ Furthermore, photonic structures can be employed to enhance photon-extraction efficiency, enabling more advanced quantum-optical characterizations such as photon indistinguishability measurements and quantum-state tomography of entangled photon pairs. Compared to GaAs QDs, a key difference is that the InGaAs QDs presented here have a higher s-p shell separation, up to a factor of 2. This could extend high-fidelity entangled photon emission from 4 K to temperatures above 40 K,^68^ allowing the operation of QDs with entanglement using cost-effective Stirling cryocoolers.^69^ Finally, the optical line widths we measured for InGaAs QDs are good but have not yet reached the Fourier-transform limit. Strategies to further improve the optical quality and to further extend the emission wavelength include replacing the AlGaAs barrier with GaAs,^70^ as this can improve optical quality by reducing impurity-related defects, and/or embedding the QDs in diode structures.^38^

## Supplementary Material



## Data Availability

The data underlying this study are openly available in Zenodo at 10.5281/zenodo.16760367.

## Notes

Growth of quantum dots (representative growth protocol) and modeling experimental data of transition energies (PDF)

## References

[1] Ekert A. K. (1991). Quantum
cryptography based on Bell’s theorem. Phys. Rev. Lett..

[2] Kimble H. J. (2008). The quantum
internet. Nature.

[3] Bennett C. H., Brassard G. (2014). Quantum cryptography:
Public key distribution and coin
tossing. Theoretical Computer Science.

[4] Wehner S., Elkouss D., Hanson R. (2018). Quantum internet:
A vision for the
road ahead. Science.

[5] Zhong H. S., Deng Y. H., Qin J., Wang H., Chen M. C., Peng L. C., Luo Y. H., Wu D., Gong S. Q., Su H., Hu Y., Hu P., Yang X. Y., Zhang W. J., Li H., Li Y., Jiang X., Gan L., Yang G., You L., Wang Z., Li L., Liu N. L., Renema J. J., Lu C. Y., Pan J. W. (2021). Phase-Programmable Gaussian Boson
Sampling Using Stimulated Squeezed Light. Phys.
Rev. Lett..

[6] Arrazola J. M., Bergholm V., Brádler K., Bromley T. R., Collins M. J., Dhand I., Fumagalli A., Gerrits T., Goussev A., Helt L. G., Hundal J., Isacsson T., Israel R. B., Izaac J., Jahangiri S., Janik R., Killoran N., Kumar S. P., Lavoie J., Lita A. E., Mahler D. H., Menotti M., Morrison B., Nam S. W., Neuhaus L., Qi H. Y., Quesada N., Repingon A., Sabapathy K. K., Schuld M., Su D., Swinarton J., Száva A., Tan K., Tan P., Vaidya V. D., Vernon Z., Zabaneh Z., Zhang Y. (2021). Quantum circuits
with
many photons on a programmable nanophotonic chip. Nature.

[7] Kwiat P. G., Waks E., White A. G., Appelbaum I., Eberhard P. H. (1999). Ultrabright source of polarization-entangled photons. Phys. Rev. A.

[8] Kuhn A., Hennrich M., Rempe G. (2002). Deterministic single-photon
source
for distributed quantum networking. Phys. Rev.
Lett..

[9] Benson O., Santori C., Pelton M., Yamamoto Y. (2000). Regulated and entangled
photons from a single quantum dot. Phys. Rev.
Lett..

[10] Ding X., Guo Y.-P., Xu M.-C., Liu R.-Z., Zou G.-Y., Zhao J.-Y., Ge Z.-X., Zhang Q.-H., Liu H.-L., Wang L.-J., Chen M.-C., Wang H., He Y.-M., Huo Y.-H., Lu C.-Y., Pan J.-W. (2025). High-efficiency
single-photon source above the loss-tolerant threshold for efficient
linear optical quantum computing. Nat. Photonics.

[11] Somaschi N., Giesz V., De Santis L., Loredo J. C., Almeida M. P., Hornecker G., Portalupi S. L., Grange T., Antón C., Demory J., Gómez C., Sagnes I., Lanzillotti-Kimura N. D., Lemaítre A., Auffeves A., White A. G., Lanco L., Senellart P. (2016). Near-optimal single-photon sources in the solid state. Nat. Photonics.

[12] Rota M. B., Krieger T. M., Buchinger Q., Beccaceci M., Neuwirth J., Huet H., Horová N., Lovicu G., Ronco G., Covre da Silva S. F., Pettinari G., Moczała-Dusanowska M., Kohlberger C., Manna S., Stroj S., Freund J., Yuan X., Schneider C., Ježek M., Höfling S., Basso Basset F., Huber-Loyola T., Rastelli A., Trotta R. (2024). A source of
entangled photons based on a cavity-enhanced and strain-tuned GaAs
quantum dot. eLight.

[13] Schweickert L., Jöns K. D., Zeuner K. D., Covre
Da Silva S. F., Huang H., Lettner T., Reindl M., Zichi J., Trotta R., Rastelli A., Zwiller V. (2018). On-demand
generation
of background-free single photons from a solid-state source. Appl. Phys. Lett..

[14] Hanschke L., Fischer K. A., Appel S., Lukin D., Wierzbowski J., Sun S., Trivedi R., Vučković J., Finley J. J., Müller K. (2018). Quantum dot
single-photon sources with ultra-low multi-photon
probability. npj Quantum Information.

[15] Arakawa Y., Holmes M. J. (2020). Progress in quantum-dot
single photon sources for quantum
information technologies: A broad spectrum overview. Applied Physics Reviews.

[16] Heindel T., Kim J.-H., Gregersen N., Rastelli A., Reitzenstein S. (2023). Quantum dots
for photonic quantum information technology. Advances in Optics and Photonics.

[17] Quandela . The power of single photon sources; Quandela Technology: 2025; accessed October 13, 2025.

[18] Sparrow Quantum . Sparrow core: Deterministic single-photon source; Sparrow Quantum Product: 2025; accessed October 14, 2025.

[19] Lodahl P. (2018). Quantum-dot
based photonic quantum networks. Quantum Science
and Technology.

[20] Dubrovskii V. G., Cirlin G. E., Ustinov V. M. (2004). The effective
thickness, temperature
and growth rate behavior of quantum dot ensembles. physica status solidi (b).

[21] Kamiya I., Tanaka I., Ohtsuki O., Sakaki H. (2002). Density and size control
of self-assembled InAs quantum dots: preparation of very low-density
dots by post-annealing. Physica E: Low-dimensional
Systems and Nanostructures.

[22] Rastelli A., Stufler S., Schliwa A., Songmuang R., Manzano C., Costantini G., Kern K., Zrenner A., Bimberg D., Schmidt O. G. (2004). Hierarchical self-assembly of GaAs/AlGaAs
quantum dots. Phys. Rev. Lett..

[23] Verma A. K., Bopp F., Finley J. J., Jonas B., Zrenner A., Reuter D. (2022). Low areal densities
of InAs quantum dots on GaAs(1
0 0) prepared by molecular beam epitaxy. J.
Cryst. Growth.

[24] Mar J. D., Xu X. L., Sandhu J. S., Irvine A. C., Hopkinson M., Williams D. A. (2010). Electrical control of fine-structure splitting in self-assembled
quantum dots for entangled photon pair creation. Appl. Phys. Lett..

[25] Schimpf C., Basset F. B., Aigner M., Attenender W., Ginés L., Undeutsch G., Reindl M., Huber D., Gangloff D., Chekhovich E. A., Schneider C., Höfling S., Predojević A., Trotta R., Rastelli A. (2023). Hyperfine
interaction limits polarization entanglement of photons from semiconductor
quantum dots. Phys. Rev. B.

[26] Zaporski L., Shofer N., Bodey J. H., Manna S., Gillard G., Appel M. H., Schimpf C., Covre da Silva S. F., Jarman J., Delamare G., Park G., Haeusler U., Chekhovich E. A., Rastelli A., Gangloff D. A., Atatüre M., Le Gall C. (2023). Ideal refocusing of an optically
active spin qubit
under strong hyperfine interactions. Nat. Nanotechnol..

[27] Huber D., Reindl M., Covre Da
Silva S. F., Schimpf C., Martín-Sánchez J., Huang H., Piredda G., Edlinger J., Rastelli A., Trotta R. (2018). Strain-Tunable GaAs Quantum Dot: A Nearly Dephasing-Free
Source of Entangled Photon Pairs on Demand. Phys. Rev. Lett..

[28] Vural H., Portalupi S. L., Michler P. (2020). Perspective of self-assembled InGaAs
quantum-dots for multi-source quantum implementations. Appl. Phys. Lett..

[29] Löbl M. C., Scholz S., Söllner I., Ritzmann J., Denneulin T., Kovács A., Kardynał B. E., Wieck A. D., Ludwig A., Warburton R. J. (2019). Excitons in InGaAs quantum dots without electron wetting
layer states. Communications Physics.

[30] Fricker D., Atkinson P., Jin X., Lepsa M., Zeng Z., Kovács A., Kibkalo L., Dunin-Borkowski R. E., Kardynał B. E. (2023). Effect
of surface gallium termination on the formation
and emission energy of an InGaAs wetting layer during the growth of
InGaAs quantum dots by droplet epitaxy. Nanotechnology.

[31] Reithmaier J. P., Sęk G., Löffler A., Hofmann C., Kuhn S., Reitzenstein S., Keldysh L. V., Kulakovskii V. D., Reinecke T. L., Forchel A. (2004). Strong coupling
in a single quantum
dot-semiconductor microcavity system. Nature.

[32] Girard J. F., Dion C., Desjardins P., Allen C. N., Poole P. J., Raymond S. (2004). Tuning of the electronic
properties of self-assembled
inas/inp(001) quantum dots by rapid thermal annealing. Appl. Phys. Lett..

[33] Rastelli A., Ulrich S. M., Pavelescu E.-M. M., Leinonen T., Pessa M., Michler P., Schmidt O. G. (2004). Self-assembled quantum dots for single-dot
optical investigations. Superlattices Microstruct..

[34] Langbein W., Borri P., Woggon U., Stavarache V., Reuter D., Wieck A. (2004). Radiatively limited
dephasing in
InAs quantum dots. Phys. Rev. B.

[35] Heyn C., Stemmann A., Köppen T., Strelow C., Kipp T., Grave M., Mendach S., Hansen W. (2009). Highly uniform and
strain-free GaAs quantum dots fabricated by filling of self-assembled
nanoholes. Appl. Phys. Lett..

[36] da
Silva S. F. C., Undeutsch G., Lehner B., Manna S., Krieger T. M., Reindl M., Schimpf C., Trotta R., Rastelli A. (2021). GaAs quantum dots grown by droplet etching epitaxy
as quantum light sources. Appl. Phys. Lett..

[37] Reindl M., Weber J. H., Huber D., Schimpf C., Covre Da
Silva S. F., Portalupi S. L., Trotta R., Michler P., Rastelli A. (2019). Highly indistinguishable single photons from incoherently
excited quantum dots. Phys. Rev. B.

[38] Zhai L., Löbl M. C., Nguyen G. N., Ritzmann J., Javadi A., Spinnler C., Wieck A. D., Ludwig A., Warburton R. J. (2020). Low-noise
GaAs quantum dots for quantum photonics. Nat.
Commun..

[39] Zhai L., Nguyen G. N., Spinnler C., Ritzmann J., Löbl M. C., Wieck A. D., Ludwig A., Javadi A., Warburton R. J. (2022). Quantum
interference of identical photons from remote GaAs quantum dots. Nat. Nanotechnol..

[40] Undeutsch G., Aigner M., Garcia A. J., Reindl J., Peter M., Mader S., Weidinger C., Covre da Silva S. F., Manna S., Schöll E., Rastelli A. (2025). Electric-Field Control
of Photon Indistinguishability in Cascaded Decays in Quantum Dots. Nano Lett..

[41] Michael C. P., Srinivasan K., Johnson T. J., Painter O., Lee K. H., Hennessy K., Kim H., Hu E. (2007). Wavelength-
and material-dependent
absorption in GaAs and AlGaAs microcavities. Appl. Phys. Lett..

[42] Michl J., Peniakov G., Pfenning A., Hilska J., Chellu A., Bader A., Guina M., Höfling S., Hakkarainen T., Huber-Loyola T. (2023). Strain-free
GaSb quantum dots as
single-photon sources in the telecom s-band. Advanced Quantum Technologies.

[43] Hakkarainen, T. ; Hilska, J. ; Hietalahti, A. ; Ranta, S. ; Peil, M. ; Kantola, E. ; Chellu, A. ; Sen, E. ; Penttinen, J.-P. ; Guina, M. Telecom wavelength single-photon source based on InGaSb/AlGaSb quantum dot technology. arxiv.org (04/09/2024), https://arxiv.org/abs/2404.06083, Accessed December 17, 2025.

[44] Yu Y., Zhong H., Yang J., Liu L., Liu J., Yu S. (2019). Highly uniform
and symmetric epitaxial InAs quantum dots embedded
inside Indium droplet etched nanoholes. Nanotechnology.

[45] Kersting E., Babin H. G., Spitzer N., Yan J. Y., Liu F., Wieck A. D., Ludwig A. (2025). Shutter-Synchronized
Molecular Beam
Epitaxy for Wafer-Scale Homogeneous GaAs and Telecom Wavelength Quantum
Emitter Growth. Nanomaterials.

[46] Spitzer N., Kersting E., Grell M., Kohminaei D., Schmidt M., Bart N., Wieck A. D., Ludwig A. (2024). Telecom o-band
quantum dots fabricated by droplet etching. Crystals.

[47] Wang L., Rastelli A., Schmidt O. G. (2006). Structural
and optical properties
of In­(Ga)­As/GaAs quantum dots treated by partial capping and annealing. J. Appl. Phys..

[48] Babiński A., Jasiński J., Bożek R., Szepielow A., Baranowski J. M. (2001). Rapid thermal
annealing of InAs/GaAs quantum dots under
a gaas proximity cap. Appl. Phys. Lett..

[49] Henini M. (1996). Molecular
beam epitaxy from research to mass-production — part 1. III-Vs Review.

[50] Zhou Q. (2013). Influence of GaAs(0 0 1) pregrowth surface morphology
and reconstruction
on the growth of ingaas layers. Appl. Surf.
Sci..

[51] da
Silva S. F. C., Mardegan T., de Araújo S. R., Ramirez C. A. O., Kiravittaya S., Couto O. D., Iikawa F., Deneke C. (2017). Fabrication and Optical Properties of Strain-free Self-assembled
Mesoscopic GaAs Structures. Nanoscale Res. Lett..

[52] Wang Y. (2019). Influence of Ga­(Al)­As
substrates on surface morphology and critical
thickness of ingaas quantum dots. Curr. Appl.
Phys..

[53] Stoffel M., Rastelli A., Stangl J., Merdzhanova T., Bauer G., Schmidt O. G. (2007). Shape oscillations: A walk through
the phase diagram of strained islands. Phys.
Rev. B.

[54] Huber D., Lehner B. U., Csontosová D., Reindl M., Schuler S., Covre da Silva S. F., Klenovský P., Rastelli A. (2019). Single-particle-picture
breakdown in laterally weakly confining GaAs quantum dots. Phys. Rev. B.

[55] Trotta R., Zallo E., Magerl E., Schmidt O. G., Rastelli A. (2013). Independent
control of exciton and biexciton energies in single quantum dots via
electroelastic fields. Physical Review B - Condensed
Matter and Materials Physics.

[56] Müller M., Bounouar S., Jöns K. D., Glässl M., Michler P. (2014). On-demand generation of indistinguishable
polarization-entangled
photon pairs. Nat. Photonics.

[57] Braun T., Betzold S., Lundt N., Kamp M., Höfling S., Schneider C. (2016). Impact of *ex situ* rapid thermal annealing
on magneto-optical properties and oscillator strength of In­(Ga)­As
quantum dots. Phys. Rev. B.

[58] Liu F., Brash A. J., O’Hara J., Martins L. M. P. P., Phillips C. L., Coles R. J., Royall B., Clarke E., Bentham C., Prtljaga N., Itskevich I. E., Wilson L. R., Skolnick M. S., Fox A. M. (2018). High purcell factor
generation of indistinguishable on-chip single photons. Nat. Nanotechnol..

[59] Liu J., Su R., Wei Y., Yao B., da Silva S. F. C., Yu Y., Iles-Smith J., Srinivasan K., Rastelli A., Li J., Wang X. (2019). A solid-state source
of strongly entangled photon pairs with high
brightness and indistinguishability. Nat. Nanotechnol..

[60] Rickert L., Vajner D. A., von Helversen M., Schall J., Rodt S., Reitzenstein S., Liu H., Li S., Ni H., Niu Z., Heindel T. (2025). High Purcell
Enhancement in Quantum-Dot Hybrid Circular
Bragg Grating Cavities for GHz Clock Rate Generation of Indistinguishable
Photons. ACS Photonics.

[61] Jahn J.-P., Munsch M., Beguin L., Kuhlmann A. V., Renggli M., Huo Y., Ding F., Trotta R., Reindl M., Schmidt O. G., Rastelli A., Treutlein P., Warburton R. J. (2016). An artificial
Rb atom in a semiconductor with lifetime-limited linewidth. Phys. Rev. B.

[62] Grange T., Ferreira R., Bastard G. (2007). Polaron relaxation
in self-assembled
quantum dots: Breakdown of the semiclassical model. Phys. Rev. B.

[63] Zibik E. A., Grange T., Carpenter B. A., Porter N. E., Ferreira R., Bastard G., Stehr D., Winnerl S., Helm M., Liu H. Y., Skolnick M. S., Wilson L. R. (2009). Long lifetimes of
quantum-dot intersublevel transitions in the terahertz range. Nat. Mater..

[64] Stobbe S., Schlereth T. W., Höfling S., Forchel A., Hvam J. M., Lodahl P. (2010). Large quantum dots with small oscillator strength. Phys. Rev. B.

[65] Peter E., Senellart P., Martrou D., Lemaître A., Hours J., Gérard J. M., Bloch J. (2005). Exciton-photon strong-coupling
regime for a single quantum dot embedded in a microcavity. Phys. Rev. Lett..

[66] Hennessy K., Badolato A., Winger M., Gerace D., Atatüre M., Gulde S., Fält S., Hu E. L., Imamoǧlu A. (2007). Quantum nature
of a strongly coupled single quantum dot-cavity system. Nature.

[67] Najer D., Söllner I., Sekatski P., Dolique V., Löbl M. C., Riedel D., Schott R., Starosielec S., Valentin S. R., Wieck A. D., Sangouard N., Ludwig A., Warburton R. J. (2019). A gated quantum dot strongly coupled
to an optical microcavity. Nature.

[68] Lehner B. U., Seidelmann T., Undeutsch G., Schimpf C., Manna S., Gawełczyk M., Covre da Silva S. F., Yuan X., Stroj S., Reiter D. E., Axt V. M., Rastelli A. (2023). Beyond the Four-Level
Model: Dark and Hot States in Quantum Dots Degrade Photonic Entanglement. Nano Lett..

[69] Schlehahn A., Krüger L., Gschrey M., Schulze J.-H., Rodt S., Strittmatter A., Heindel T., Reitzenstein S. (2015). Operating
single quantum emitters with a compact stirling cryocooler. Rev. Sci. Instrum..

[70] Wang Z. M., Holmes K., Shultz J. L., Salamo G. J. (2005). Self-assembly of
GaAs holed nanostructures by droplet epitaxy. physica status solidi (a).

