# Supplementary material for: Low-Density InGaAs/AlGaAs Quantum Dots in Droplet-Etched Nanoholes
Source: Nano Lett. 2026 Jan 15;26(3):953–60. doi: 10.1021/acs.nanolett.5c04426 (PMC12856885; doi:10.1021/acs.nanolett.5c04426)
Supplement: Supplementary file 1 [file nl5c04426_si_001.pdf]

# Supporting Information: Low-Density InGaAs/AlGaAs Quantum Dots in Droplet-Etched Nanoholes


Saimon F. Covre Da Silva,[*,†,‡] Ailton J. Garcia Jr,[†] Maximilian Aigner,[†] Christian Weidinger,[†] Tobias M. Krieger,[†] Gabriel Undeutsch,[†] Christoph Deneke,[‡] Ishrat Bashir,[¶] Santanu Manna,[†,¶] Melina Peter,[†] Ievgen Brytavskyi,[†] Johannes Aberl,[†] and Armando Rastelli[†]

†*Institute of Semiconductor and Solid State Physics, Johannes Kepler University Linz, Altenberger Straße 69, 4040 Linz, Austria*

‡*Instituto de Física Gleb Wataghin, Universidade Estadual de Campinas (UNICAMP), 13083-859 Campinas, Brazil*

¶*Department of Electrical Engineering, Indian Institute of Technology Delhi, Hauz Khas, New Delhi, Delhi 110016, India*

E-mail: saimon@unicamp.br


## 1 Quantum dot growth and properties

The samples were grown by molecular beam epitaxy (MBE) using a *MBE Komponenten — Dr. Eberl* system equipped with standard effusion cells for Ga, In, and Al, and a valved cracker cell for $As_4$. The growth was carried out on quarters of 2”-diameter n-doped GaAs (001) substrates, which were desorbed at a substrate temperature (measured by the thermocouple placed behind the substrate) $T_{\mathrm{deox}}$(= 569 C in the example shown in Table S1) under an As overpressure and subsequently annealed at $T_{\mathrm{deox}} + 21\,°\mathrm{C}$ for 10 minutes. A 40 nm GaAs

buffer layer was then grown to planarize the surface at the same substrate temperature, with surface quality assessed by reflection high-energy electron diffraction (RHEED). After that, we determine the temperature $T_{\mathrm{ave}}$ at which the transition from a GaAs (4×4) to (2×4) surface reconstruction occurs, identified by asymmetric RHEED streaks along the [110] and [$1\bar{1}0$] directions. This temperature, which corresponds to a real temperature of about 500 °C is used as a reference for the subsequent growth steps. The substrate temperature was then increased to $T_{\mathrm{ave}} + 94$ °C, and an additional 100 nm GaAs buffer layer was grown, followed by a 30-period AlAs/GaAs superlattice (2.5 nm/2.5 nm) to improve the surface quality and bury possible impurities and defects. Prior to the growth, a beam equivalent pressure (BEP) of $3.0 \times 10^{-5}$ mbar for $As_4$ was measured.

In the next step, a 200 nm-thick $Al_{0.30}Ga_{0.70}As$ bottom barrier was deposited (line 5 in Table S1). Subsequently, the As cracker valve was completely closed over a 10-second interval. With the As flux fully suppressed, an amount of Al corresponding to 0.5 monolayers (ML) of AlAs was deposited at a growth rate of 0.50 µm h$^{-1}$ (line 7). Optimizing the etching time is crucial for achieving high-quality quantum dots. After the Al deposition, a 20-second

Table S1: Example growth report for the sample with $In_{0.4}Ga_{0.6}As$-filled QDs

| Step | Comment | Repeats | Material | Thickness (nm) | Duration (s) | Sub. TC (C) | Pyrometer (C) | PMBE (mbar) |
|---|---|---|---|---|---|---|---|---|
| 1 | | | GaAs buffer | 50,000 | 360,0 | 590 | 594 | 2,46E-06 |
| 2 | Superlattice | 30 | AlAs | 2,500 | 18,0 | 590 | 594 | 2,50E-06 |
| 3 | Superlattice | | GaAs | 2,500 | 18,0 | 590 | 597 | 2,31E-06 |
| 4 | Superlattice | | GI | 0,000 | 5,0 | 590 | 5594 | 2,70E-06 |
| 5 | | | AlGaAs | 200,000 | 964,8 | 590 | 598 | 2,19E-06 |
| 6 | Close As | | GI | 0,000 | 10,0 | 590 | 594 | 6,58E-07 |
| 7 | Al droplet | | Al | 0,1415 | 1,0 | 590 | | |
| 8 | Droplet coalescence | | GI | 0,000 | 20,0 | 590 | 592 | 3,01E-07 |
| 9 | etching | | GI | 0,000 | 60,0 | 590 | 591 | 5,04E-07 |
| 10 | etching | | GI | 0,000 | 60,0 | 590 | 591 | 6,28E-07 |
| 11 | crystallization | | GI | 0,000 | 60,0 | 590 | 592 | 2,77E-06 |
| 12 | sub ramp | | GI | | 150,0 | 495 | | 3,05E-06 |
| 13 | filling | 1 | In.40Ga0.60As | 1,000 | 6,1 | 495 | 506 | 2,97E-06 |
| 14 | | | | | | | 496 | 3,05E-06 |
| 15 | | | GI | | 30,0 | 495 | 498 | 3,14E-06 |
| 16 | filling | | GaAs cap | 0,144 | 1,0 | 495 | 500 | 3,12E-06 |
| 17 | | | GI | | 30,0 | 495 | 506 | 3,16E-06 |
| 18 | sub ramp | | GI | | 120,0 | 590 | | 3,13E-06 |
| 19 | | | AlGaAs | 200,000 | 964,8 | 590 | 608 | 2,33E-06 |
| 20 | Close As | | GI | 0,000 | 10,0 | 590 | 597 | 2,19E-07 |
| 21 | Al droplet | | Al | 0,1415 | 1,0 | 590 | | 4,00E-07 |
| 22 | Droplet coalescence | | GI | 0,000 | 20,0 | 590 | 587 | 2,77E-07 |
| 23 | etching | | GI | 0,000 | 60,0 | 590 | 587 | 5,10E-07 |
| 24 | etching | | GI | 0,000 | 60,0 | 590 | 587 | 6,32E-07 |
| 25 | crystallization | | GI | 0,000 | 60,0 | 590 | 589 | 2,82E-06 |
| 26 | sub ramp | | GI | | 150,0 | 495 | | 3,09E-06 |
| 27 | filling | | In.40Ga0.60As | 1,000 | 7,0 | 495 | 511 | 2,97E-06 |
| 28 | | | GI | | 30,0 | 495 | 495 | 3,01E-06 |

annealing step (still with the As valve closed) was performed to allow Al droplets to form and initiate the local etching of the underlying AlGaAs. LDE requires re-exposing the surface to an As flux. For our MBE system, optimal results were obtained by gradually increasing the As BEP from $3.0 \times 10^{-7}$ to $3.0 \times 10^{-6}$ and finally to $2.5 \times 10^{-5}$ mbar in 1-minute steps.

After nanohole formation, the substrate temperature was decreased to $T_{\mathrm{ave}}$ within 150 s. Next, a 1 nm-thick layer of $\mathrm{In}_x\mathrm{Ga}_{1-x}\mathrm{As}$ was deposited, followed by a 30 s growth interruption to promote the diffusion of $\mathrm{In}_x\mathrm{Ga}_{1-x}\mathrm{As}$ into the nanoholes. Afterwards, 0.5 ML (0.14 nm) of GaAs were grown to limit In desorption at high temperatures. Subsequently, the substrate temperature was ramped up to $T_{\mathrm{deox}} + 21\,°\mathrm{C}$ and a top $\mathrm{Al}_{0.30}\mathrm{Ga}_{0.70}\mathrm{As}$ layer was deposited, completing the optically active structure: $\mathrm{Al}_{0.30}\mathrm{Ga}_{0.70}\mathrm{As}$ / $\mathrm{In}_x\mathrm{Ga}_{1-x}\mathrm{As}$ / $\mathrm{Al}_{0.30}\mathrm{Ga}_{0.70}\mathrm{As}$.

The entire process was then restarted (line 19), this time omitting the top $\mathrm{Al}_{0.30}\mathrm{Ga}_{0.70}\mathrm{As}$ layer. This allowed atomic force microscopy (AFM) characterization of the filled nanoholes, as shown in Figure 1(c, d) of the main text. The growth process was carried out under substrate rotation. Because of the short deposition time of Al for droplet formation (1s) compared to the time duration of a full substrate rotation (10 rpm), some radial and azimuthal gradients can be expected. This condition can, in principle, lead to some radial inhomogeneity across larger wafers; however, given the reduced sample size (quarter of 2" wafer), these effects are small, as demonstrated by PL imaging performed in different areas.

An important factor to achieve highly symmetric QDs is the substrate etching temperature. In our experiments, we used a relatively high temperature, which resulted in asymmetric nanohole shapes. Optimizing this step is therefore crucial for obtaining QDs with low FSS. Finally we note that all samples contain QDs with nominally the same shape and size, which is determined by the nanohole and top barrier, but different stoichiometry.

## 1.1 Density mapping via photoluminescence imaging

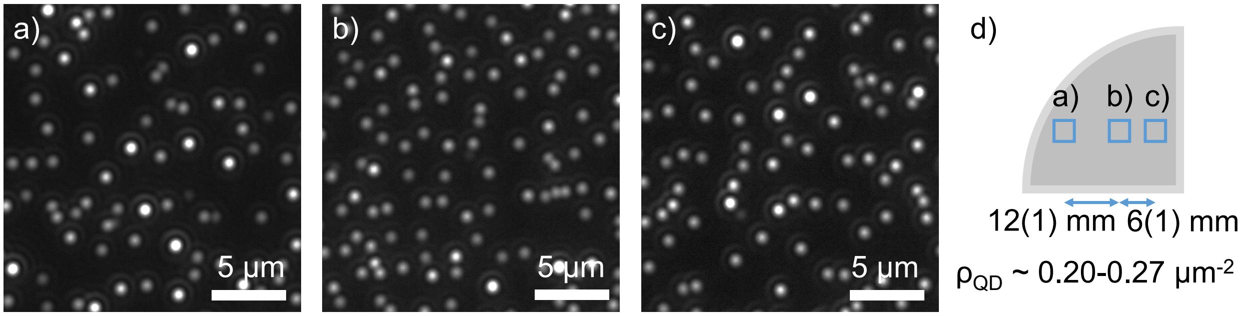


Figure S1: **Optical image density mapping.** $(20 \times 20)$ µm$^2$ large images of (a) region I, with 81 QDs, (b) region II, with 108 QDs, and (c) region III, with 81 QDs. (d) Schematic illustration of the distances between the regions on the quarter wafer.

The density of QDs was assessed using the sample with an In content of $x = 0.3$. The sample was illuminated with blue light from a LED centered at 470 nm and the spectrally filtered PL emission of the QDs was imaged on a camera sensor using a 800 nm long-pass filter. Images of three different pieces of different locations but from the same sample, i.e., a quarter of a 2" wafer, were taken and are shown in Fig. S1 a)-c). The approximate origin of those pieces is shown in d). To assess the QD density, QDs within $(20 \times 20)$ µm$^2$ regions were manually counted and the density was found to vary between 0.20(2) and 0.27(3) µm$^{-2}$, where the quoted uncertainties assume Poissonian counting statistics.

## 1.2 Emission wavelength distribution

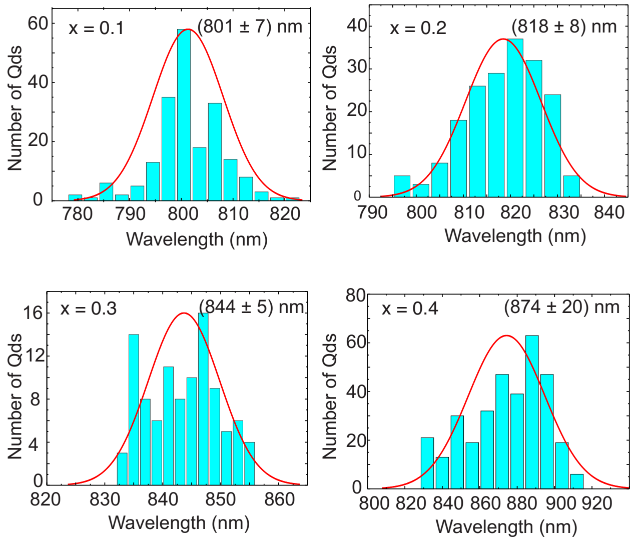


Figure S2: **Wavelength histogram distribution for the InGaAs/AlGaAs QDs.**

Figure S2 depicts histograms of the wavelength distribution for samples with $x = 0.1$, 0.2, 0.3, and 0.4. For $x = 0.4$, the standard deviation is noticeably larger, possibly indicating indium inhomogeneity during the nanohole filling process.

## 1.3 Fine Structure Splitting of a representative QD

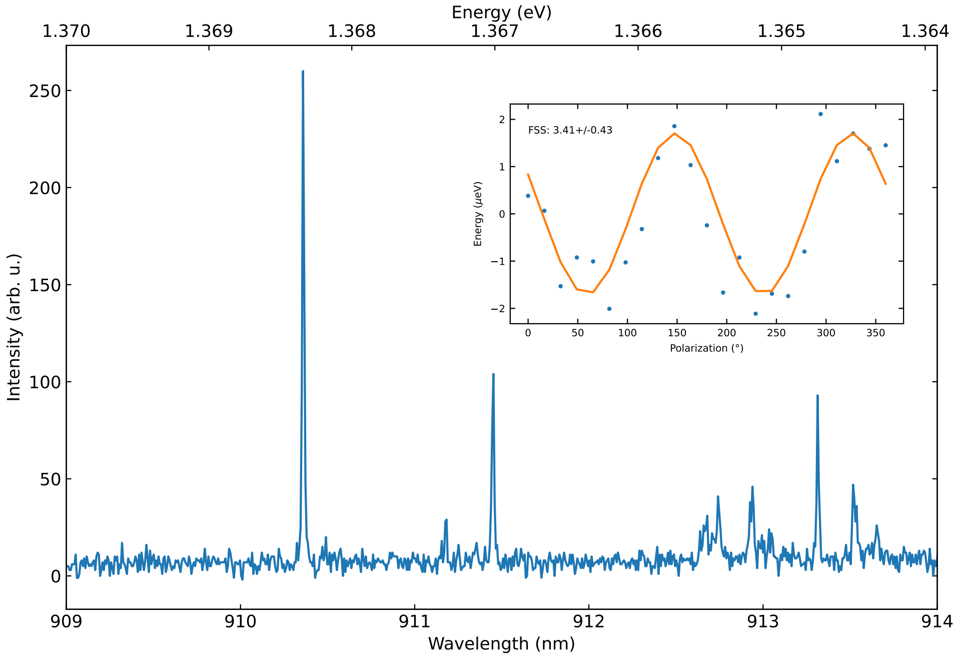


Figure S3: **Spectrum and fine structure splitting for a QD in the sample with** $x = 0.4$. The inset shows the position of the maximum of a gaussian fit of the neutral exciton line as a function of the orientation of the polarization analyzer on the collection path. By fitting the resulting plot with a cosine function we extract a FSS of 3.4(4) μeV.

## 1.4 Polarization-resolved PL measurement

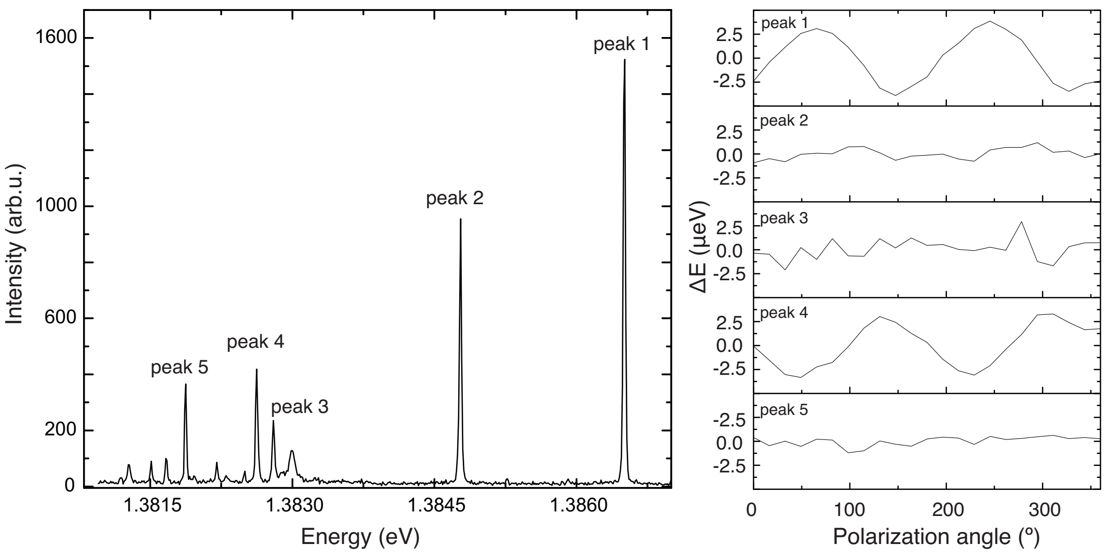


Figure S4: **Left:** µPL spectrum of a single QD from the sample with $x = 0.4$. **Right:** Polarization analysis for the 5 most intense emission peaks, where $\Delta E = 0$ corresponds to the averaged position of each peak.

Figure S4 show that peaks 2, 3 and 5 exhibit no measurable polarization dependence, as expected for charged exciton transitions. In contrast, the neutral exciton and biexciton transitions (peak 1 and 4) display the characteristic sinusoidal polarization splitting, associated with the fine-structure splitting.

## 1.5 Temperature-dependent PL measurements

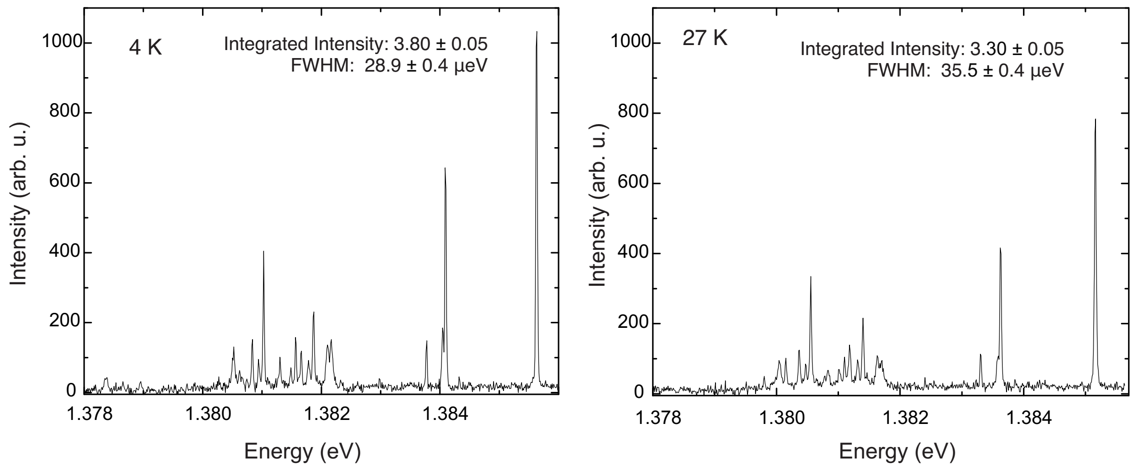


Figure S5: Spectra of a single QD from the samples with $x = 0.4$ for 4 K and 27 K.

Figure S5 shows PL spectra of the same QD emission for two different temperatures. Fitting the emission with a Gaussian yields the neutral exciton's integrated intensity and full width at half maximum (FWHM). Both linewidths are below the resolution limit of $\sim 40$ µeV. For this temperature range, the integrated intensity decreases by approximately 13%.

# 2 Modeling and experimental correlation

The actual composition and resulting emission properties of InGaAs filled nanoholes are governed by complex kinetic and thermodynamic processes during growth. To gather some insight in the alloy composition of the QDs presented in this work, we use the optical spectroscopy data and compare them to calculation results using two modeling approaches: (i) the Single Band Model (SBM) and (ii) the more rigorous 8-band k.p approach. We focus on the ground-state transition energy and we model the QDs as simple QWs with thickness corresponding the nanohole depth (QD height). This approximation is justified by the fact that the QDs are shallow (about 8 nm high and about 50-60 nm wide), so that the confinement energies are dominated by the QD height. From the observed s-p shell separation, lateral confinement contributes about 20 meV to the electron confinement energy. However, we expect this contribution to be partly compensated by the exciton binding energy, which is not included in our model.

Figure S1(a) compares the ground-state transition energies predicted by SBM and 8-band k.p models as a function of In composition, both with and without strain. We note that we limited the range of In composition to values corresponding to the range of experimentally observed transition energies. In the explored composition range, the SBM model predicts slightly lower transition energies compared to the multi-band model. In both models, inclusion of compressive strain (due to lattice mismatch between InGaAs and AlGaAs lattice matched to GaAs) results in a blue shift of the transition energy compared to the unstrained case. This is expected, as strain raises the conduction band and splits/lifts the valence band, effectively widening the optical bandgap. Furthermore, we have carried out 3D simulations for conical quantum dots (QDs) with an 8 nm height and basal diameters of 60 nm and 120 nm to evaluate the effect of three-dimensional confinement. The results show a blue shift in the transition energy by ∼35 meV (corresponding to ∼20 nm in wavelength) and ∼20 meV (∼10 nm in wavelength), respectively, compared to the 1D QW approximation.

Figure S1(b) compares the nominal In composition $x$ used in the MBE growth recipe (x axis) with the In content in a homogeneously alloyed QW (left y axis) which would produce the experimentally observed emission energy (right axis, red data points).

The data suggest that there is a linear relation between expected and nominal In contents, but that the expected values are substantially lower than the nominal ones. For example, when the nominal In fraction $x$ is 40%, the effective In content within the QD is only ∼15%. This significant discrepancy highlights key non-idealities during nanohole filling. The reduced incorporation of In can be qualitatively attributed to surface segregation. Due to segregation, In accumulates at the growth surface during deposition and is desorbed during the heating

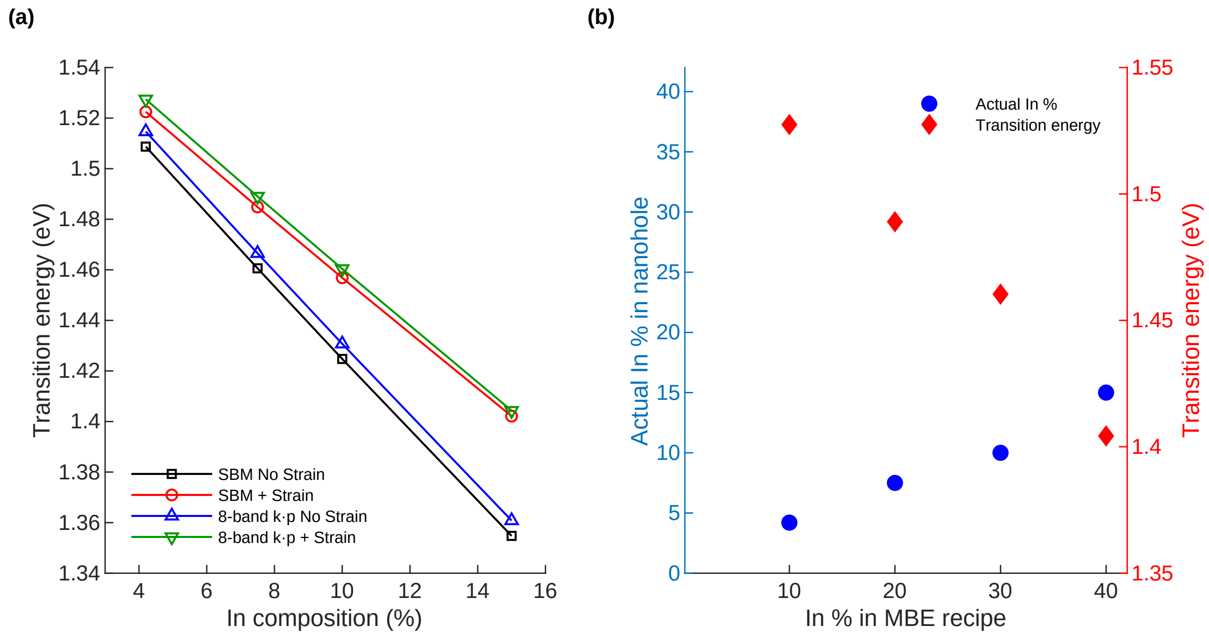


Figure S6: **Calculated impact of strain and alloy composition on InGaAs QW/QD emission.** (a) Comparison of interband transition energies computed using the single-band model and the 8-band k.p model, both with and without strain. The quantum well structure is used as an effective model for a strained InGaAs QD embedded in $\mathrm{Al}_{0.33}Ga_{0.67}\mathrm{As}$. The inclusion of strain consistently leads to a blue-shift in the transition energy for both models. (b) Correlation between the nominal In composition used in MBE growth (x-axis) and the estimated average In incorporation inside the nanohole (left y-axis), deduced from the experimental emission energy (right y-axis). The discrepancy between nominal In content $x$ and theoretical average composition suggests that only part of the deposited In is actually incorporated in the QDs.

step preceding the growth of the top AlGaAs barrier. At present we are not able to establish whether the In distribution in the nanoholes is inhomogeneous. Detailed structural studies would be required to assess this point.
